# Evaluation of a hydroalcoholic extract of liquorice (*Glycyrrhiza glabra* L.) root on the treatment of experimentally induced peritonitis in New Zealand male rabbits

**DOI:** 10.17221/31/2025-VETMED

**Published:** 2025-12-31

**Authors:** Mohammad Ahmadi, Iraj Nowrouzian, Mostafa Norbakhsh, Mehrdad Yadegari, Mohsen Jafarian Dehkordi

**Affiliations:** ^1^Department of Veterinary Clinical Sciences, Shk.C., Islamic Azad University, Shahrekord, Iran; ^2^Department of Veterinary Clinical Sciences, Faculty of Veterinary Medicine, University of Tehran, Tehran, Iran; ^3^Department of Clinical Pathology, Shk.C., Islamic Azad University, Shahrekord, Iran

**Keywords:** liquorice extract, peritonitis, rabbits, treatment

## Abstract

This experimental study evaluated the therapeutic potential of a liquorice-derived hydroalcoholic extract in managing experimentally induced peritonitis in New Zealand rabbits. The animals were randomly divided into six groups (*n* = 6): one control group, one negative control group (infected but untreated), three treatment groups that received the liquorice-derived extract at 0.5, 1, and 2 g/kg, and one positive control group treated with enrofloxacin. The confirmation of peritoneal infection relied on histopathological and radiographic tests. The effect of the liquorice extract on the peritoneal infection was evaluated using biochemical, haematological, and ultrasound analyses across the groups. The ultrasound examination, along with the haematological and biochemical evaluation on the 20^th^ day after induction, showed significant differences between the groups. The results demonstrated that higher doses of 1 g/kg and 2 g/kg were more effective than the lower dose of 0.5 g/kg. The haematological and biochemical analyses revealed significant differences in several variables (including WBC, neutrophils, fibrinogen, and liver enzymes) between the treated and control groups, with the most pronounced improvements observed in the group receiving 2 g/kg of liquorice extract, suggesting a dose-dependent therapeutic effect. The administration of a hydroalcoholic extract of liquorice at different doses, along with the standard treatment with enrofloxacin, affected various haematological and biochemical variables in the context of peritoneal infection management. In conclusion, the effectiveness of the liquorice-derived extract is dose-dependent and could be used as an effective therapeutic agent in peritoneal infections in New Zealand rabbits. These findings showed that the liquorice-derived extract effectively improves the local inflammatory and structural changes associated with peritoneal infection without adversely affecting systemic biochemical homeostasis.

Inflammation is a complex biological response to harmful stimuli, from which the term is derived from the Latin root meaning “to burn” (Abdulkhaleq et al. 2018). The inflammatory mechanism involves organised and dynamic responses, including cellular events, vascular changes, and humoral secretions, ultimately leading to the synthesis of immune cells and angiogenic factors (Huether and McCance 2015). The creation of new vessels helps maintain the inflammation by facilitating the migration of inflammatory cells and extracellular matrix molecules to the site of injury (Marme and Fusenig 2008; Ribatti and Crivellato 2009).

Peritoneal infections, or inflammation of the thin layer covering the abdominal cavity and organs, are a biological reaction involving blood vessels, immune cells, and inflammatory mediators in response to injury or microbial infections (Kalliolias and Ivashkiv 2016).

Clinical manifestations often include abdominal pain, infection, and potentially multiple organ failure, with mortality rates exceeding 60% (Li et al. 2023; Huang et al. 2024). Although the underlying pathogenic mechanisms are not fully understood, there is a clear connection between peritoneal infections and white blood cells, signalling molecules (prostaglandins, histamine, leukotrienes, free radicals), and cytokines such as tumour necrosis factor-alpha (TNF)-α and interleukin-6 (IL-6) (Idriss and Naismith 2000; Anwikar and Bhitre 2010; Huether and McCance 2015).

The current therapeutic approaches for peritoneal infection include a combination of medical management (glucocorticoids, immunosuppressants, and antibiotics) and surgical intervention, depending on the underlying cause and severity (Buijk and Bruining 2002; Li et al. 2022). While standard management is effective in many cases, challenges remain regarding adverse effects, the optimisation of antibiotic regimens, and the management of complications, highlighting the need for complementary therapeutic approaches (Huang et al. 2024).

Liquorice (*Glycyrrhiza glabra* L.) is a natural sweetener and one of the oldest herbal medicines, with documented use in ancient Assyria, Egypt, China, and India (Armanini et al. 2002). Its traditional uses include the treatment of swelling, stomach ulcers, respiratory infections, and as an antitussive (Kim et al. 2015; Vyas et al. 2017; Pastorino et al. 2018). Liquorice is recognised as a food additive by the United States Food and Drug Administration, the Council of Europe, and the Joint FAO/WHO Committee (FAO/WHO 2006; Pastorino et al. 2018).

The therapeutic effects of liquorice are attributed to its main compounds, including liquiritin, isoliquiritin, glycyrrhizin, liquiritigenin, isoliquiritigenin, 18β-glycyrrhetinic acid, liquiritin apioside, glycyrrhetic acid, licochalcone A, and glabridin (El-Saber Batiha et al. 2020). These compounds exhibit antioxidant, anti-inflammatory, analgesic, immunomodulatory, anti-diabetic, antimicrobial, and regulatory properties for the digestive system.

Liquorice-derived compounds, particularly glycyrrhizin and glycyrrhetinic acid, are relevant to peritoneal infection treatment due to their potent anti-inflammatory and immunomodulatory effects. These compounds can reduce inflammation by targeting pathways such as NF-κB and MAPK, and by inhibiting the production of inflammatory cytokines. Their antimicrobial properties further aid in controlling infections. The liquorice extract also inhibits inflammatory pathways, reduces oxidative stress, regulates cytokines, induces interferon, enhances immune cell activity, and reduces pathogen burden in the peritoneal cavity (Lakshmi and Geetha 2011; Kim et al. 2015; Nazari et al. 2017; Kuang et al. 2018).

This study aimed to evaluate the therapeutic effects of a liquorice-derived hydroalcoholic extract on an *E. coli*-induced peritoneal infection in male New Zealand rabbits, focusing on haematological, biochemical, imaging, and histopathological outcomes. We also specifically investigated the dose-dependent efficacy of a liquorice extract at concentrations of 0.5, 1, and 2 g/kg to determine the optimal therapeutic dose for managing peritoneal inflammation.

## MATERIAL AND METHODS

### Animals

The current study was an experimental laboratory intervention conducted at the Rabbit Laboratory of Islamic Azad University, Shahrekord Branch, Iran.

The total of thirty-six male New Zealand white rabbits weighing 2.0–2.5 kg, aged 4–5 months, were housed in standard cages under controlled conditions (20 °C, 45% humidity, 12-hour light-dark cycle). The animals were acclimatised for one week before the start of the experiments. All the procedures were conducted in accordance with the Animal Research: Reporting of *In Vivo* Experiments (ARRIVE) guidelines for reporting animal research. All the experimental protocols were approved by the Animal Care Ethics Committee of the Islamic Azad University, Shahrekord Branch (Approval No: IR.IAU.SHK.REC.1402.008, Date: January 15, 2023).

### Preparation of liquorice-derived hydroalcoholic extract

The liquorice plant, scientifically identified as *Glycyrrhiza glabra* L. was harvested from Kian, a locality in Shahrekord within the Chaharmahal and Bakhtiari Province of Iran (32.284719° N, 50.894594° E), in January 2024, and transported to a medicinal plant research facility. Upon arrival, the root was authenticated by the facility’s botanist and assigned a herbarium identification number. The root was then cleaned, dried, and ground into a powder.

This powdered liquorice was subjected to hydroalcoholic extraction: the powdered root was soaked in 70% ethanol (Razi, Isfahan, Iran) for 72 h at ambient temperature. The mixture was filtered every 24 h using Whatman No. 1 filter paper (Whatman; GE Healthcare, Maidstone, UK), and fresh solvent was added to the residue for further extraction. The combined extracts were then concentrated through vacuum distillation (IKA, Staufen, Germany) and further dried in an oven at temperatures below 40 °C to ensure stability and potency.

### Treatments

The study consisted of six groups: Group 1: Control group (no medication), Group 2: Negative control (*E. coli* bacterial suspension), Group 3: *E. coli* + 0.5 g/kg liquorice extract, Group 4: *E. coli* + 1 g/kg liquorice extract, Group 5: *E. coli* + 2 g/kg liquorice extract, Group 6: Positive control (*E. coli* + enrofloxacin 5–10 mg/kg i.m. every 12 hours).

All the treatments were administered on days 0, 1, 3, and 7 following the *E. coli* inoculation.

### Rationale for dose selection

The doses of 0.5, 1, and 2 g/kg were selected based on preliminary dose-finding studies and previous literature on liquorice extract in rabbit models (Nazari et al. 2017; Kuang et al. 2018). These doses were determined to be within the therapeutic range while avoiding potential toxicity. Preliminary studies indicated that doses below 0.5 g/kg showed minimal therapeutic effects, while doses above 2 g/kg began to demonstrate potential adverse effects on the gastrointestinal motility in rabbits.

### Animal monitoring and welfare

All the rabbits were monitored daily for behavioural changes, appetite, and general condition. The body weights were recorded on days 0, 7, 14, and 20. No significant weight loss (>10%) or abnormal behaviour was observed in any group, indicating that the experimental procedures were well tolerated.

### Determination of phenol and flavonoid of liquorice-derived hydroalcoholic extract

The total phenolic content was determined using the Folin–Ciocalteu method. Ten (10) to 20 mg of the prepared ethanolic extract was dissolved in 60% methanol to achieve a 10 ml volume. Then, 0.01 ml of this solution was mixed with 0.5 ml of the 10% Folin–Ciocalteu reagent (Sigma-Aldrich, St. Louis, USA) and 4 ml of a 7.5% sodium carbonate solution (Nasr, Tehran, Iran). The tubes were kept at laboratory temperature for 30 min, and the absorbance was measured at 755 nm using a spectrophotometer (Shimadzu, Kyoto, Japan). The total phenols were determined using a standard curve based on mg of gallic acid (Sigma-Aldrich, St. Louis, USA) per gram of extract.

The total flavonoid content was measured by dissolving 10 mg to 20 mg of the extract in 60% methanol to a final volume of 10 ml. One ml of this solution was mixed with 1 ml of a 2% aluminium chloride solution (Nasr, Tehran, Iran), and the absorbance was read after 40 min at 415 nm in three replications. The flavonoid content was determined based on the equivalent amount of quercetin (Sigma-Aldrich, St. Louis, USA) in each gram of the extract.

### Induction of the peritoneal infection in rabbits using *E. coli*

**Figure 1:**
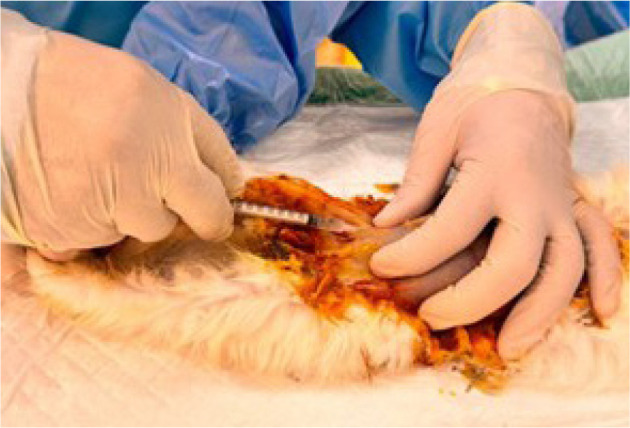
Induction of the peritoneal infection with an *Escherichia coli* bacterial suspension containing 2 × 10^8^ cells/ml

A post-procedure ultrasound confirmed the correct administration in all the cases, ensuring the accurate delivery of the bacterial suspension into the peritoneal cavity. By establishing a controlled peritoneal infection model using *E. coli* in rabbits, we were able to investigate the efficacy of the liquorice-derived product and study the underlying mechanisms of infection. An *E. coli* bacteria suspension containing 2 × 10^8^ cells per millilitre (cells/ml) was prepared in normal saline (Nasr, Tehran, Iran). Rabbits were put under general anaesthesia to ensure they were pain-free and immobile during the procedure (Figure 1).

The rabbits’ abdomen was shaved to remove the fur, which helped prevent contamination during the procedure. Antiseptic solutions, especially povidone-iodine (Pasargad, Noor, Iran), followed by alcohol (Pasargad, Noor, Iran), were applied to the surgical site to reduce the risk of infection from external sources. A sterile syringe with gauge 27 (Helma Teb, Tehran, Iran) was used to inject 0.5 ml of the *E. coli* suspension into the abdominal cavity of the rabbit (Figure 1).

### Ultrasound evaluation of the peritoneal infection

**Figure 2:**
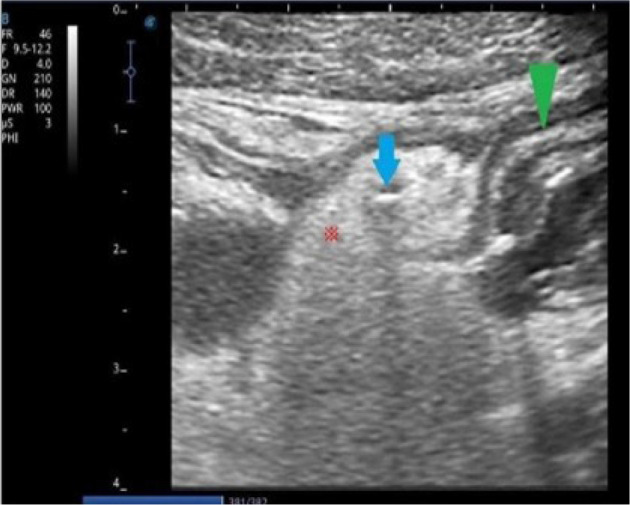
Ultrasound confirmation of the peritoneal infection 4 days after inoculation (red star: high intensity peritoneal echo; blue arrow: free gas; green arrowhead: thickening of the intestinal wall)

**Figure 3:**
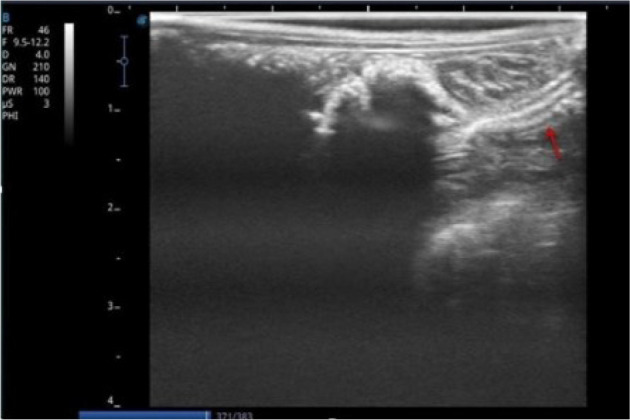
Normal peritoneal ultrasound (red arrow: normal small intestine wall, lower abdominal fat echo with hypo-echo spots)

**Figure 4:**
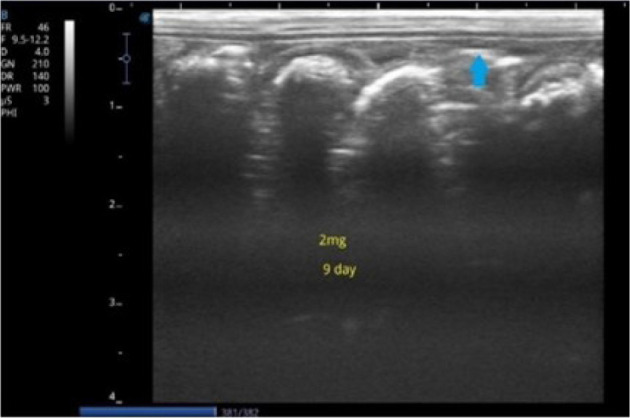
Ultrasound findings of a peritoneal infection in the 2 g/kg treatment group (blue arrow: the wall thickness of the small intestine was normal and free echogenic particles were not observed, free gas was not observed, continuous hyperechoic lines between the peritoneum were completely eliminated)

**Figure 5:**
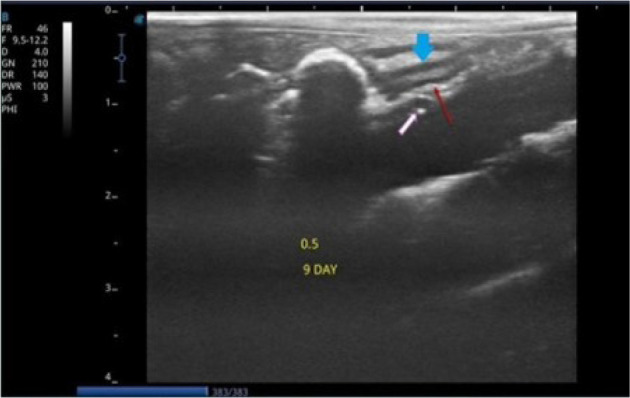
Ultrasound findings of a peritoneal infection in the 0.5 g/kg treatment group (blue arrow: the increase in the thickness of the small intestine wall is still observed – the presence of echogenic particles in the ventricular area is evident; red arrow: the irregular border of visceral organs can be recognised)

**Figure 6:**
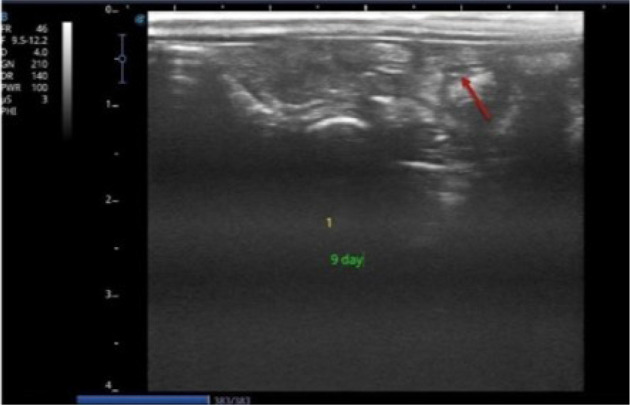
Ultrasound findings of peritonitis in the 1 g/kg treatment group (arrow: the thickness of the small intestine wall decreased compared to the control group and the 0.5 g/kg liquorice extract treatment group, the amount of echogenic particles in the ventricular area decreased compared to the control group, the free gas of the abdominal area decreased compared to the control group)

To minimise gastrointestinal motility, facilitate anaesthesia, and enhance experimental outcomes, each animal was fasted for 12 h immediately before the ultrasound examination. Although fasting is not typically recommended for rabbits due to the risk of gastrointestinal stasis, a 12-hour fasting period was implemented prior to the ultrasound examination to ensure the optimal visualisation of the abdominal structures. This short duration was deemed acceptable as it is within a safe fasting window for rabbits undergoing brief procedures (Harcourt-Brown 2008). To induce anaesthesia, a combination of ketamine (Alfasan, Woerden, the Netherlands) at a dose of 60 mg/kg and medetomidine (Alfasan, Woerden, the Netherlands) at a dose of 100 μg/kg was administered intramuscularly. To prepare the animals for the ultrasound evaluation, the animals’ body hair was carefully shaved at the midline location from approximately 1 centimetre (cm) posterior to the xiphoid cartilage to the posterior part of the pubic region. Then, the animal was placed on its back, and an ultrasound examination was performed by pouring gel on the skin surface using a Medison EX8000 ultrasound machine with a frequency of 7–9 megahertz (MHz) from a proper abdominal approach. A complete ultrasound examination was performed with the same ventral approach in each rabbit. An ultrasound was performed one time on the 9^th^ day between the extract injection and control groups, and at the end, the ultrasound evaluation process and findings are detailed with confirmation of the peritoneal infection shown in Figure 2, the normal finding in Figure 3, the 2 g/kg treatment group results in Figure 4, the 0.5 g/kg treatment group results in Figure 5, and the 1 g/kg treatment group results in Figure 6.

### Measuring the haematological and biochemical indicators

After the treatment period, blood was drawn through the auricular vein, and the samples were sent to the laboratory for haematology and biochemical tests. Haematology factors including the red blood cell count (RBC), white blood cell count (WBC), haemoglobin (Hb), packed cell volume (PCV), neutrophils (Nut), lymphocytes (Lym), monocytes (Mon), band cells (Band), eosinophils (Eos), basophils (Baso), and fibrinogen (Fibrino) were measured using an automated blood cell counter (Table 1).

**Table 1:** Measuring the haematological indicators

Parameters	PCV	RBC	Hb	WBC	Nut	Lym	Mon	Eos	Baso	Band	Fibrino
*P*-value	<0.001	<0.001	0.010	<0.001	<0.001	<0.001	<0.001	0.326	0.857	<0.001	<0.001

Band = band cell; Baso = basophils; Eos = eosinophils; Fibrino = fibrinogen; Hb = haemoglobin; Lym = lymphocytes; Mon = monocytes; Neut = neutrophils; PVC = packed cell volume; RBC = red blood cell; WBC = white blood cell

Daily behavioural observations and body weight measurements were recorded throughout the study period. The 2 g/kg treatment group showed significantly less lethargy and better appetite compared to the negative control group (*P* < 0.05). The body weight changes demonstrated that the 2 g/kg group maintained their weight better than the negative control group, with a mean weight loss of only 3.2% compared to 8.7% in the negative control group (*P* < 0.01).

To evaluate the liver function, the levels of alanine aminotransferase (ALT), aspartate aminotransferase (AST), alkaline phosphatase (ALP), cholesterol (CHO), total protein (T.Pr), and albumin (Alb) were measured. To evaluate kidney function, the levels of urea, creatinine (Cr), blood urea nitrogen (BUN), and gamma-glutamyltransferase (GGT) were investigated. The data obtained from the haematology and biochemical tests were analysed using appropriate statistical software (Table 2). Considering the significance of the *P*-value in most haematology parameters, more extensive statistical tests are required to assess the magnitude of the difference between the different groups in this research. Comparisons between groups were performed using the Kruskal–Wallis non-parametric statistical test for independent samples or with an analysis of variance (ANOVA).

**Table 2:** Measuring the biochemical indicators

Parameters	BUN	Crea	CHO	T.Pr	Alb/ Serum	Alb/ Globulin	AST	ALT	ALP	GGT	Urea serum
*P*-value	0.174	0.171	0.486	0.692	0.078	0.402	0.028	0.017	0.070	0.113	0.673

Alb = albumin; Alb/Globulin = albumin in globulin, Alb/Serum = albumin in serum; ALP = alkaline phosphatase; ALT = alanine aminotransferase; AST = aspartate aminotransferase; BUN = blood urea nitrogen; CHO = cholesterol; Crea = creatinine; GGT = gamma-glutamyl transferase; T.Pr = total protein

### Data analysis

The SPSS v21 software was used for the statistical analysis of the biochemical and haematological results. The data were analysed using a one-way analysis of variance and expressed as the mean ± standard error of the mean. If the difference was statistically significant (*P* ≤ 0.05), pairwise group comparisons were performed.

### Ethical approval

All the experimental protocols were approved by the Animal Care Ethics Committee of the Islamic Azad University, Shahrekord Branch. Ethical Approval Code Ref. No: IR.IAU.SHK.REC. 1402.008.

## RESULTS

### Ultrasound evaluation of the peritoneal infection

This study compared different doses of the liquorice-derived extract in the treatment of a peritoneal infection. The higher doses of 1 g/kg and 2 g/kg were more effective than the lower dose of 0.5 g/kg. The group treated with 1 g/kg exhibited significant symptom improvement, including a decrease in the small intestine wall thickness, echogenic particles, and free gas in the abdominal area.

However, the group treated with 2 g/kg showed complete improvement in symptoms, with a normal thickness of the small intestine wall, no echogenic particles or free gas, and the disappearance of hyperechoic lines between the peritoneum (Figure 4).

### Radiography results – Radiographic findings of the peritoneal infection

The radiographic evaluation of the rabbits diagnosed with a peritoneal infection revealed several important findings. The observed complications included a loss of peritoneal detail associated with decreased contrast between the fat and soft tissue opacity, abdominal distention due to the accumulation of liquid or gas (appearing as areas of increased soft tissue opacity), free fluid or gas expansion, and a mottled or streaky opacity pattern, indicating local inflammation or fluid accumulation.

### Histopathology results

**Figure 7:**
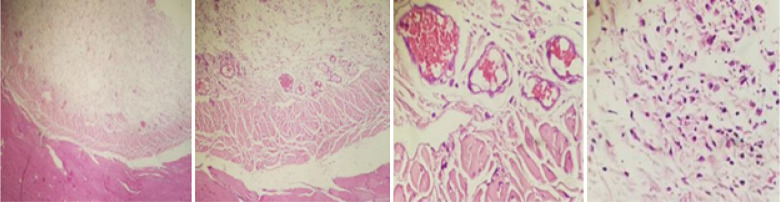
The three layers of muscles were healthy, but the outer serous covering (adventitia) was dense and showed signs of bleeding, with infiltration of inflammatory cells, especially lymphocytes, plasma cells, and neutrophils

The histopathological examination of the rabbit treatment with the liquorice-derived extract for a peritoneal infection revealed two distinct outcomes:

Negative control group (Group 2): Examination of the abdominal wall tissues revealed three healthy muscular layers, along with the adventitia or serosa. Although the muscular layers were intact, the adventitia showed signs of congestion and haemorrhage, with the infiltration of inflammatory cells, including lymphocytes, plasma cells, and neutrophils. These findings indicate the presence of inflammation and resultant tissue damage, likely due to the peritoneal infection (Figure 7).

**Figure 8:**
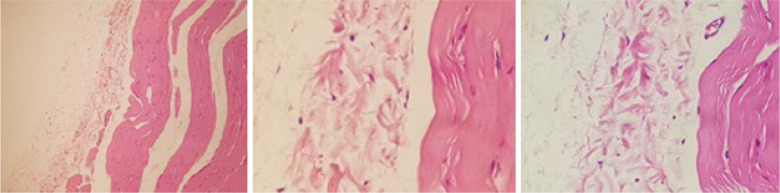
Three layers of muscle were healthy, with no signs of necrosis or inflammatory cell infiltration. The adventitia layer showed no signs of inflammation

Treatment groups (Groups 3–6): The abdominal wall tissues in these groups displayed three healthy muscular layers with no signs of necrosis or inflammatory cell infiltration. The adventitia was also devoid of any inflammation and appeared completely clean. These observations suggest that the treatments administered in these groups were effective in controlling the inflammation and preventing the tissue damage caused by the peritoneal infection (Figure 8).

In summary, the histopathological results from the control group (first group) indicate an inflammatory condition and tissue damage in response to a peritoneal infection. In contrast, the outcomes from the treatment group (the second group) demonstrate improvement and control of inflammation, preventing tissue damage due to the efficacy of the therapeutics, including antibiotics and a liquorice-derived extract.

### Haematology and biochemical results

The results of the haematological tests were evaluated with the Kruskal–Wallis test, and the following results were obtained (Table 3).

**Table 3:** Independent samples of the Kruskal–Wallis test summary for haematological and biochemical traits

Indicators	Total *N*	Test statistic	Degree of freedom	Asymptotic significance (2-sided test)	Significant differences between groups
PCV	36	21.859^a^	5	<0.001	1–2/2–4
RBC	36	22.362^a^	5	<0.001	1–2/2–4/2–3
Hb	36	15.170^a^	5	0.010	1–3
WBC	36	24.816^a^	5	<0.001	1–3/1–2/2–6
Neut	36	27.722^a^	5	<0.001	1–3/1–2/2–6/2–5
Lym	36	29.504^a^	5	<0.001	1–2/2–6/3–6/1–3
Mon	36	22.343^a^	5	<0.001	1–2/1–4/2–6/4–6
Band	36	27.869^a^	5	<0.001	1–2/1–4/2–6/4–6
Fibrinogen	36	30.757^a^	5	<0.001	2–6/3–6/1–3/1–2/5–2
AST	36	12.586^a^	5	0.028	2–4
ALT	36	13.826^a^	5	0.017	1–4

ALT = alanine aminotransferase; AST = aspartate aminotransferase; Band = band cell; Fibrino = fibrinogen; Hb = haemoglobin; Lym = lymphocytes; Mon = monocytes; Neut = neutrophils; PVC = packed cell volume; RBC = red blood cell; WBC = white blood cell

The summarised results show how the different treatments, including isoliquiritigenin at various doses and the standard treatment with enrofloxacin, affect the haematological and biochemical indicators. The 2 g/kg dose demonstrated statistically significant improvements in multiple indicators (*P* ≤ 0.05) compared to the control groups, indicating its superior effectiveness in managing peritoneal infections.

## DISCUSSION

A peritoneal infection is an internal inflammation in the peritoneal space that can be acute, chronic, focal, or diffuse, posing serious health risks. In the present study, the effects of a hydroalcoholic extract of liquorice root on improving the peritoneal infection in New Zealand male rabbits were investigated, and it was found that the liquorice-derived extract improves the peritoneal infection. There was a statistically significant difference in the 2 g/kg liquorice-derived hydroalcoholic extract group compared to the other groups, as indicated by the haematological and biochemical results.

Although the precise mechanism of action of the liquorice-derived extract in the treatment of the peritoneal infection was not the primary aim of this study, it has been shown that liquorice root contains glycyrrhizin or glycyrrhizinic acid. Glycyrrhizin is a vasoconstrictor molecule with corticosteroid-like activities and is metabolised to other compounds after oral administration (Wang et al. 2015; Rizzato et al. 2017; Pastorino et al. 2018). Oral liquorice with or without glycyrrhizin is used in many herbal medicines for its medicinal activities and also for its taste and sweetness (Hasan et al. 2021).

Compounds such as glycyrrhizin and 18-β-glyc-yrrhetinic acid, present in liquorice, affect hormonal activity by inhibiting cortisol metabolism and reducing testosterone synthesis (Dissanayake et al. 2020). In laboratory studies, liquorice has also demonstrated anti-obesity, blood fat reduction, liver protection, anti-spasmodic, and anti-asthmatic properties (Kao et al. 2014; Ombelt and Van Robays 2015). Additionally, liquorice has hepatoprotective and antioxidant activities that prevent reactive oxygen species and lipid peroxidation in liver cells. In general, liquorice and its compounds have a wide range of therapeutic effects, making them valuable in the treatment of various conditions (Lakshmi and Geetha 2011).

Our findings regarding the anti-inflammatory effects of isoliquiritigenin are consistent with those of Pastorino et al. (2018), who demonstrated that a liquorice-derived extract reduces inflammatory markers in rodent models of peritonitis by inhibiting the NF-κB pathway. Similarly, Lakshmi and Geetha (2011) reported that isoliquiritigenin specifically targets the TNF-α and IL-6 production, which aligns with our observed reductions in the inflammatory parameters. However, our study extends these findings by demonstrating dose-dependent efficacy in a rabbit model of *E. coli*-induced peritonitis and providing comprehensive imaging and histopathological evidence of tissue recovery.

The radiographic assessments revealed several critical abnormalities associated with the peritoneal infection, including loss of peritoneal detail, abdominal distention, and patterns indicative of local inflammation or fluid accumulation. These observations are consistent with the previous literature, which characterises the peritoneal infection through similar radiographic features, emphasising the severity and complexity of this condition (Li et al. 2022; Huang et al. 2024). The resolution of these radiographic abnormalities, particularly with higher doses of liquorice, underscores its therapeutic potential in restoring a standard abdominal architecture, which is a crucial aspect of peritoneal infection recovery.

The ultrasound findings further corroborated the radiographic evidence, demonstrating significant improvements in the structural integrity of the abdominal cavity with increasing doses of liquorice-derived extract. Notably, the highest dose (2 g/kg) resulted in normalisation of the small intestine wall thickness and the resolution of echogenic particles and free gas, indicating a substantial reduction in inflammation and fluid accumulation. The dose-dependent efficacy observed suggests that the liquorice-derived extract shows its therapeutic effects through mechanisms that are enhanced at higher concentrations. This phenomenon warrants further investigation to elucidate the underlying pharmacodynamics.

Haematological and biochemical variables showed significant differences between liquorice-derived extract treatment groups and the control groups. These findings suggest that the liquorice-derived extract effectively addresses the local inflammatory and structural changes associated with the peritoneal infection without adversely affecting the systemic haematological and biochemical homeostasis. This activity of the liquorice-derived extract is encouraging, as it involves a broader therapeutic approach with minimal systemic side effects, which is a crucial consideration in the management of peritoneal infections.

Enrofloxacin is widely used as a first-line antibiotic in the treatment of bacterial peritonitis in veterinary medicine (Li et al. 2022 – ISPD Peritonitis Guideline Recommendations 2022). However, our study demonstrates that liquorice-derived extract, particularly at a dose of 2 g/kg, shows comparable or superior efficacy in certain parameters, suggesting its potential as an alternative or complementary therapy.

The dose-dependent efficacy of liquorice-derived extract demonstrated in this study suggests its potential as an adjunctive therapy for peritonitis, particularly in cases of antibiotic resistance or when minimising antibiotic use is desirable. Given the safety profile of the liquorice extract and its lack of adverse systemic effects, it could be incorporated into multimodal treatment protocols for peritonitis, potentially reducing the antibiotic doses and associated side effects. Future clinical trials should investigate optimal dosing regimens and potential synergies with conventional antibiotics in larger animal models before translation to human medicine.

In conclusion, our study highlights the promising therapeutic role of a hydroalcoholic extract of liquorice in treating peritoneal infections, with high-dose administration (2 g/kg) yielding the most significant improvement in disease manifestations. The data presented here elucidate the dose-dependent efficacy of a liquorice-derived extract and reinforce the importance of comprehensive diagnostic evaluations in assessing treatment outcomes.

Our findings demonstrate that the liquorice-derived extract effectively improves the local inflammatory and structural changes associated with peritoneal infections without adversely affecting the systemic biochemical homeostasis. The most pronounced improvements were observed in the group receiving 2 g/kg of the liquorice extract, suggesting a dose-dependent therapeutic effect.

The translational potential of our findings is significant, particularly in the context of antibiotic resistance. The dose-dependent efficacy suggests that liquorice-derived extract compounds could be used as an adjuvant therapy to reduce the antibiotic doses. Future clinical studies should investigate optimal delivery methods and dosing regimens for human applications, particularly in patients undergoing peritoneal dialysis who are at high risk for peritoneal infections.
